# Neonatal Choledocholithiasis: A Rare but Challenging Surgical Scenario

**DOI:** 10.7759/cureus.93518

**Published:** 2025-09-29

**Authors:** Eden S Singh, Ruby Lopez-Flores, Steven Thornton, Fengming Chen, Debra Sudan, Deepak Vikraman Sushama, Tamara Fitzgerald, Emily Greenwald

**Affiliations:** 1 Department of Surgery, Duke University School of Medicine, Durham, USA; 2 Department of Pathology, Duke University Medical Center, Durham, USA; 3 Department of Surgery, Duke University Medical Center, Durham, USA; 4 Department of Pediatrics, Duke University School of Medicine, Durham, USA

**Keywords:** choledocholithiasis, general pediatric surgery, hyperbilirubinemia, infant health, roux-en-y hepaticojejunostomy

## Abstract

Choledocholithiasis is a rare diagnosis in neonates, and there are no standard management guidelines for this population. Additionally, endoscopic retrograde cholangiopancreatography (ERCP) is often not an option due to the small size of the common bile duct and non-availability of appropriately sized neonatal endoscopes. We present the cases of two neonates with choledocholithiasis. Both patients underwent open cholecystectomy with common bile duct exploration. The biliary stones were not removable, and therefore both underwent a Roux-en-Y hepaticojejunostomy. Both neonates have done well postoperatively. While hyperbilirubinemia is common in neonates, choledocholithiasis is challenging to diagnose. Diagnosis requires a broad differential and the use of abdominal ultrasound. While ERCP is the gold standard in adults, it is difficult to perform in neonates. Therefore, other treatment modalities involving laparoscopic and open surgery may be necessary. There is a need for improved management guidelines and endoscopic treatment options for neonates with choledocholithiasis.

## Introduction

Cholelithiasis is a frequent finding in the adult population but is rare in the neonatal period. When present, it may be associated with total parenteral nutrition, diuretic therapy, sepsis, congenital heart disease, prematurity, hemolysis, or cephalosporin antibiotic treatment [1]. Cholelithiasis can progress to choledocholithiasis, a condition where a gallstone is present in the common bile duct. It typically presents with jaundice, biliary colic, dark urine, and acholic stools [1,2]. Hyperbilirubinemia in the neonatal period is common. Conjugated hyperbilirubinemia can be due to a wide range of anatomic abnormalities, metabolic, infectious, and genetic disorders [3]. Surgical indications for the management of conjugated hyperbilirubinemia are anatomic abnormalities, including choledochal cysts, choledocholithiasis, or biliary atresia [4,5]. In this report of two cases, we discuss the work-up for and management of neonatal hyperbilirubinemia and specifically describe cases where the hyperbilirubinemia is the result of choledocholithiasis.

Choledocholithiasis in neonates is rare, with an estimated incidence of less than one in 5000 [6]. Occurring much more frequently in adults, choledocholithiasis is treated either with endoscopic cholangiopancreatography (ERCP), which is a minimally invasive procedure done under fluoroscopic and direct imaging that can remove gallstones that are present within the common bile duct [7]. This is typically followed by laparoscopic cholecystectomy which is an operation that removes the gallbladder. An alternative is intraoperative common bile duct (CBD) exploration with attempted clearance during cholecystectomy. If ERCP is not successful in adults, patients will typically undergo a surgical intervention or endoscopic procedure, such as temporary biliary stenting [2].

Unfortunately, there are no standard guidelines regarding the management of choledocholithiasis in infants. Management strategies described in the literature have varied, including ERCP with sphincterotomy or papillary balloon dilation, open common bile duct exploration with cholecystectomy, and laparoscopic common bile duct exploration with cholecystectomy. Other less commonly used strategies include endoscopic treatment, cholecystectomy and observation, percutaneous transhepatic drainage, nutritional optimization, and ursodeoxycholic acid therapy [6]. While ERCP is an important therapeutic treatment in adults and older children, it can be difficult to perform in neonates due to the small size of the CBD [8]. There are pediatric duodenoscopes available with an outer diameter of 7.5-7.6 mm and a working diameter of 2 mm that are intended to be used in children less than 10-15 kg or younger than 12 months. For biliary or pancreatic therapy, adult duodenoscopes are preferred as biliary dilation balloons and stents greater than 5F size are not able to be used through the 2-mm channel. However, there are stone retrieval balloons and baskets that can be utilized with the 2-mm channel [9].

We present a case series of two neonates with choledocholithiasis who were unable to undergo ERCP. The neonates were taken for open cholecystectomy with CBD exploration, and both neonates had unremovable CBD stones. Therefore, they each had a Roux-en-Y hepaticojejunostomy performed. A Roux-en-Y hepaticojejunostomy creates a biliary-enteric drainage pathway by creating an anastomosis between a defunctionalized limb of jejunum and the common bile duct [10]. These cases highlight the management of choledocholithiasis in infants who are unable to undergo ERCP and have unsuccessful open CBD exploration. It also underscores the need for improved equipment availability and management guidelines for treating choledocholithiasis in infants.

## Case presentation

Patient one

The patient was a nine-month-old male neonate, born at 40 weeks with a past medical history notable for jaundice after birth that resolved without intervention by two months of age. Family history was significant for a maternal grandmother with cholelithiasis. There was no known family history of bleeding or clotting disorders, jaundice, or splenectomy. The patient presented to an urgent care center with a one-day history of bilious emesis. At that time, he was producing wet diapers but had not had a bowel movement since the onset of emesis. On physical exam, he appeared well-hydrated, without jaundice or notable findings on abdominal exam. He was diagnosed with gastroenteritis, observed to be tolerating oral intake without emesis, and was subsequently discharged with anti-emetic therapy. Three days later he presented to the pediatric emergency department with persistent bilious emesis and a one-day history of acholic stools, raising concern for hyperbilirubinemia. The patient was afebrile with a heart rate of 160 beats per minute, respiratory rate of 34 breaths per minute, and an oxygen saturation of 97%. On physical exam, the patient was fussy, had anicteric sclera, and his abdomen was soft, non-tender, and non-distended.

Complete blood count was significant for leukocytosis and microcytic anemia. Lactate dehydrogenase (LDH) and gamma glutamyl transferase (GGT) were elevated. His total bilirubin, conjugated bilirubin, liver enzyme tests, lipase, hemoglobin electrophoresis, lead level and iron studies were all within normal limits (Table 1).

**Table 1:** Patient One's Significant Laboratory Values Notable laboratory values for patient one from presentation, three months post-operation, and five months post-operation. Reference values included.

Timeline	Laboratory Value Type	Patient One's Value	Reference Value
Presentation			
	White blood cell count	14.3 x 10^9/L	3.8-14.0 x 10^9/L
	Hemoglobin	9.0 g/dL	10.0-18.0 g/dL
	Lactate Dehydrogenase	483 U/L	160-300 U/L
	Gamma Glutamyl Transferase	214 U/L	5-18 U/L
3 months Post-Operation			
	Lactate Dehydrogenase	387 U/L	160-300 U/L
	Hemoglobin	10.1 g/dL	10.0-18.0 g/dL
5 months Post-Operation			
	Hemoglobin	9.9 g/dL	10.0-18.0 g/dL
	Aspartate Aminotransferase (AST)	48 U/L	8-30 U/L

**Figure 1:**
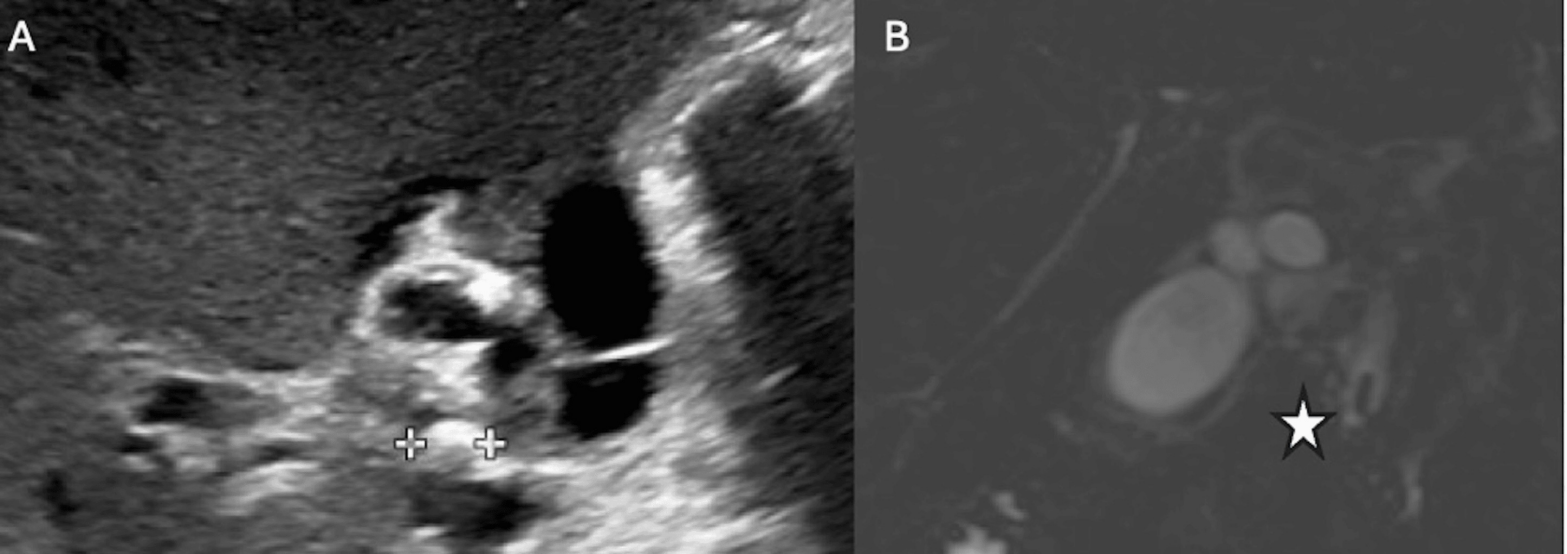
Biliary imaging for case presentation one A) Abdominal ultrasound showing a gallstone within the cystic duct. Crosshairs indicate the width of the gallstone. B) Magnetic resonance cholangiopancreatography showing a 6 mm stone within the common bile duct. The star demonstrates the location of the stone.

An ultrasound was performed that showed pericholecystic fluid as well as multiple echogenic foci within the gallbladder (Figure 1A).

Since the patient had bilious emesis, an upper gastrointestinal contrast study was performed that demonstrated normal rotation. The patient was admitted to the pediatric surgical service. On hospital day one, magnetic resonance cholangiopancreatography (MRCP) was performed, showing a distal CBD stone and extrahepatic ductal dilation consistent with choledocholithiasis (Figure 1B). Interestingly, his admission total bilirubin and conjugated bilirubin did not align with this finding. On hospital day two, the liver enzymes increased. 

**Figure 2:**
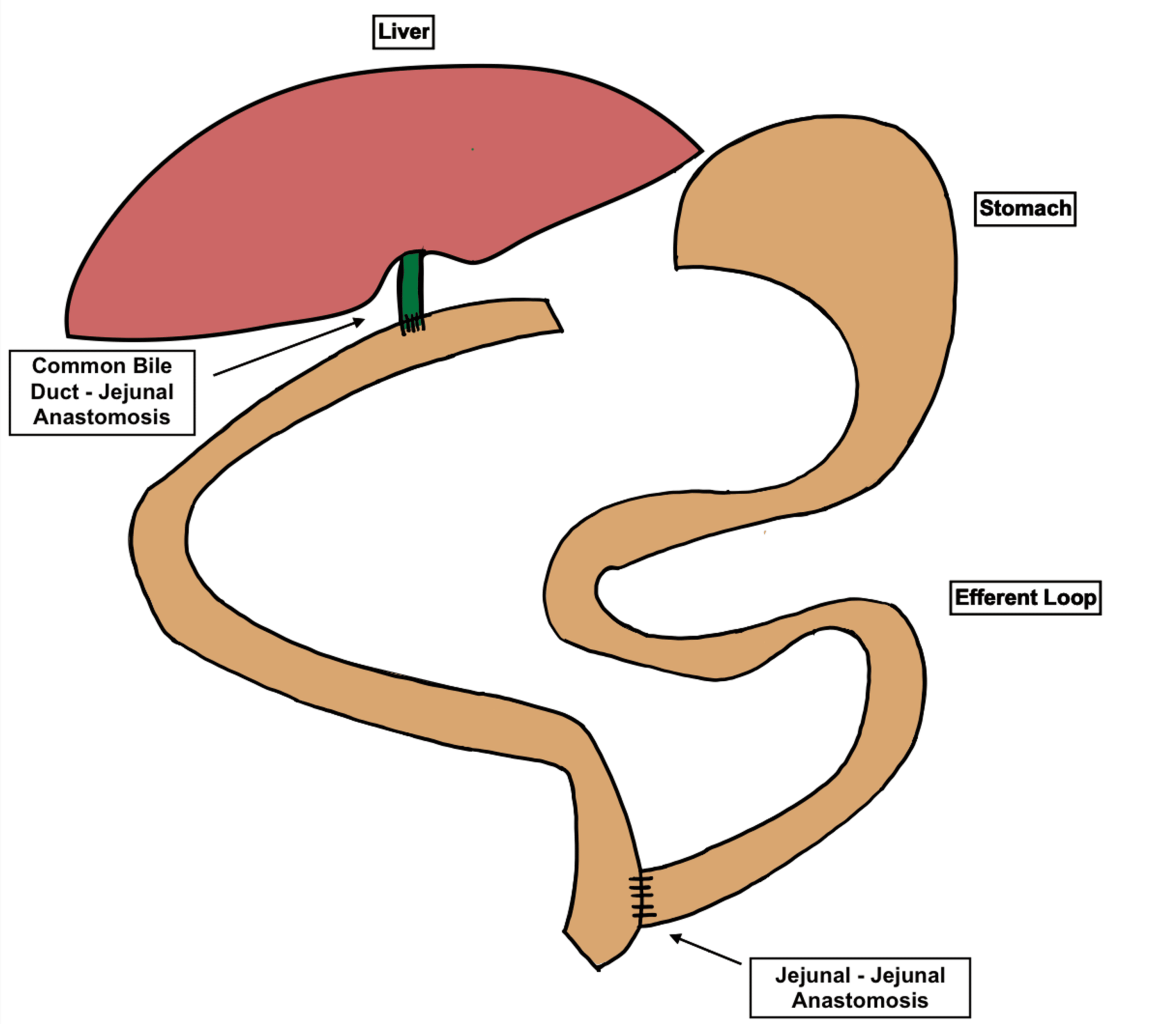
Diagram of Roux-En-Y Hepaticojejunostomy Image created by the authors.

Due to the patient’s size, ERCP was not available, and he was taken to the operating room. The gallbladder was found to be distended, with a torturous and inflamed cystic duct. Open cholecystectomy was performed. Intraoperative cholangiogram illuminated the right and left hepatic ducts, but contrast did not pass into the duodenum, indicating obstruction. Due to the small size of the CBD a standard choledochoscope could not be passed, and various endoscopes were used to examine the CBD, including a pediatric bronchoscope and a 3mm laparoscope. The stone was identified in the outlet of the CBD. The CBD was irrigated and instrumented with dilators, balloon catheters, and stone forceps but the stone was unable to be removed. Repeat cholangiogram confirmed ongoing obstruction. Additional attempts to dislodge the stone were unsuccessful, so a Roux-en-Y hepaticojejunostomy was performed in standard fashion (Figure 2). The liver was biopsied, and a portal lymph node was removed for pathology.

**Figure 3:**
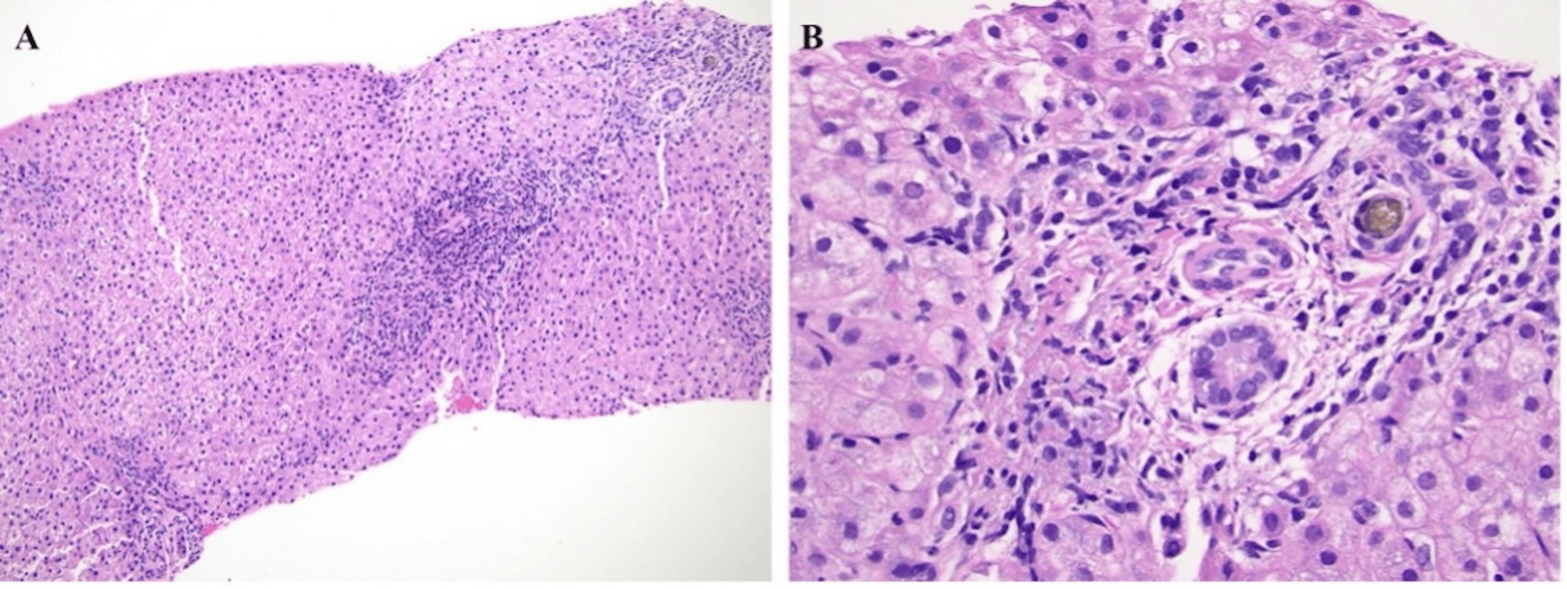
Histological findings for case presentation one suggest bile duct obstruction secondary to choledocholithiasis. (A, 10x) Liver biopsy shows portal mixed acute and chronic inflammation accompanied by bile ductular reactions. (B, 40x) Focal portal edema and ductular cholestasis are identified.

The final pathology showed a benign portal lymph node and a gallbladder with signs of chronic cholecystitis and reactive fibrosis. The liver biopsy demonstrated mild portal inflammation, no significant lobular inflammation or steatosis, and mild periportal fibrosis by trichrome stain consistent with choledocholithiasis (Figure 3).

The patient did well in the initial post-operative period. He required a blood transfusion on postoperative day three for a hemoglobin of 6.5 g/dL, but his course was otherwise unremarkable. He was started on sips of clears on post-operative day three. He was advanced to a regular diet and discharged on postoperative day eight with a three-month course of trimethoprim-sulfamethoxazole [11]. Three weeks after surgery, he was afebrile, eating and stooling normally. Three months postoperatively, the patient’s LDH had decreased and his hemoglobin had increased (Table 1). Genetic testing was performed and demonstrated a variant of unknown significance in EHHADH, which encodes for an enzyme located in the liver and kidney that is involved in the peroxisomal beta-oxidation pathway. Mutations in EHHADH are associated with Zellweger spectrum disorder [12], which has been associated with cholestasis and hepatic dysfunction [13].

Five months postoperatively, the patient re-presented to the emergency department with 24 hours of nonbilious vomiting, decreased oral intake, and abdominal pain. His last bowel movement was two days prior, raising concern for bowel obstruction. Labs were notable for a mildly low hemoglobin and an elevated aspartate aminotransferase (AST) (Table 1). The patient had findings suggestive of a possible partial small bowel obstruction on computed tomography distal to the Roux-en-Y anastomosis, and a nasogastric tube was placed for decompression. A gastrografin challenge was performed, which demonstrated contrast reaching the colon, indicating resolution of the possible partial small bowel obstruction. He was discharged on hospital day two after he had a bowel movement and was tolerating his usual diet. The patient has been referred to hematology for continued anemia and is pending further work-up. 

Patient two

The patient was a two-month-old male neonate, born at 38 weeks, whose delivery was complicated by a nuchal cord and low birth weight in the 1st percentile. He presented to his pediatrician with a two-week history of lethargy. Review of systems revealed several days of acholic stools and dark urine. Family history was significant for hyperbilirubinemia in his older sister which resolved with phototherapy. Laboratory testing demonstrated an elevated total bilirubin and alanine aminotransferase (ALT). Complete blood count was significant for leukocytosis and thrombocytosis (Table 2). 

**Table 2:** Patient Two's Significant Laboratory Values Notable laboratory values for patient two from presentation, the emergency department, hospital day eight, and post-operation. Reference values included.

Timeline	Laboratory Value Type	Patient Two's Value	Reference Value
Presentation			
	Total Bilirubin	5.1 mg/dL	0.4-1.5 mg/dL
	Alanine Aminotransferase (ALT)	91 U/L	5-45 U/L
	White blood cell count	13.6 x 10^9/L	3.8-14 x 10^9/L
	Platelets	472 x 10^9/L	150-400 x 10^9/L
Emergency Department			
	Aspartate Aminotransferase (AST)	77 U/L	8-30 U/L
	Alanine Aminotransferase (ALT)	86 U/L	5-45 U/L
	Total Bilirubin	5.7 mg/dL	0.4-1.5 mg/dL
	Conjugated Bilirubin	4.1 mg/dL	0.1-0.6 mg/dL
	Gamma Glutamyl Transferase	737 U/L	518 U/L
	C-reactive Protein	1.86 mg/dL	< 0.6 mg/dL
	White blood cell count	22.7 x 10^9/L	3.8-14 x 10^9/L
	Platelets	819 x 10^9/L	150-400 x 10^9/L
Hospital Day Eight			
	Aspartate Aminotransferase (AST)	68 U/L	8-30 U/L
	Alanine Aminotransferase (ALT)	54 U/L	5-45 U/L
	Total Bilirubin	4.2 mg/dL	0.4-1.5 mg/dL
	Conjugated Bilirubin	2.7 mg/dL	0.1-0.6 mg/dL
Hospital Follow-up Appointment			
	Aspartate Aminotransferase (AST)	104 U/L	8-30 U/L
	Alanine Aminotransferase (ALT)	75 U/L	5-45 U/L
	Total Bilirubin	5.6 mg/dL	0.4-1.5 mg/dL
	Conjugated Bilirubin	3.6 mg/dL	0.1-0.6 mg/dL
Post-operation			
	Aspartate Aminotransferase (AST)	44 U/L	8-30 U/L
	Alanine Aminotransferase (ALT)	66 U/L	5-45 U/L
	Total Bilirubin	1.8 mg/dL	0.4-1.5 mg/dL
	Conjugated Bilirubin	1.0 mg/dL	0.1-0.6 mg/dL

Due to these findings, the pediatrician recommended evaluation in the emergency department.

In the emergency department, he was afebrile with a heart rate of 151 beats per minute, respiratory rate of 33 breaths per minute, and a blood pressure of 74/60 mmHg. Physical exam was significant for mild bilateral scleral icterus and mild jaundice to the nipple line. There were no significant findings on abdominal examination. Laboratory testing was significant for an elevated AST, ALT, total bilirubin, conjugated bilirubin, GGT, C-reactive protein (CRP), and increased leukocytosis and thrombocytosis (Table 2). 

**Figure 4:**
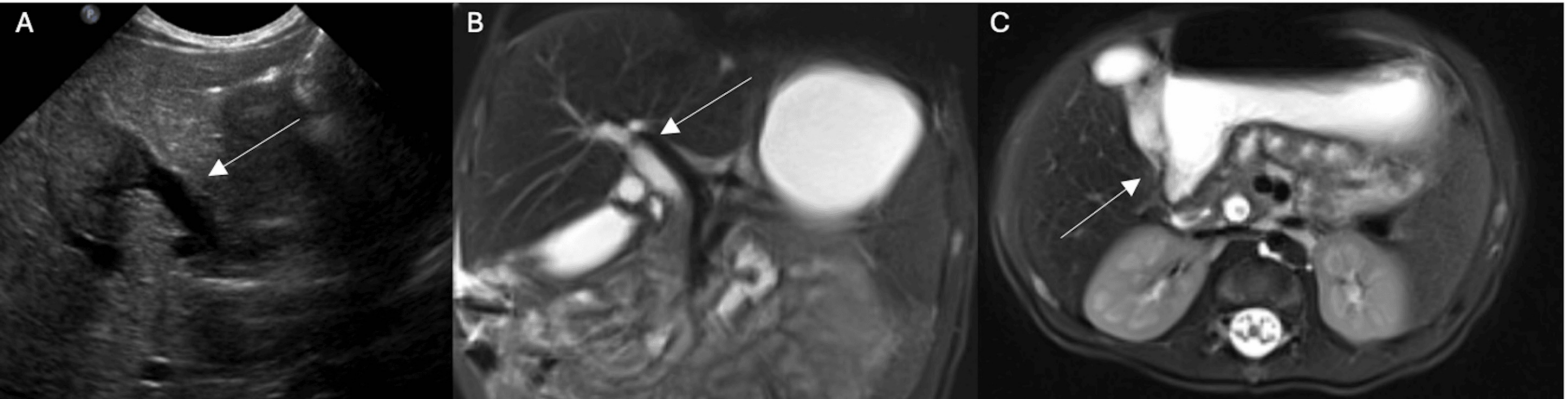
Biliary imaging for case presentation two A) Liver ultrasound showing common bile duct (CBD) and gallbladder dilation. Arrow highlights distension. B) Magnetic Resonance Cholangiopancreatography (MRCP) demonstrating dilation of the common hepatic and CBD. Arrow highlights the dilation. C) MRCP demonstrating a filling defect in the distal CBD. Arrow demonstrates filling defect.

There were no abnormalities of the thyroid profile, internal normalized ratio (INR), partial thromboplastin time (PTT), or urinalysis. Bloodwork for alpha-1-antitrypsin, Epstein-Barr virus, and cytomegalovirus (CMV) was negative. On liver ultrasound, there were no visible gallstones. The CBD and gallbladder were dilated, raising concern for distal obstruction (Figure 4A).

Due to the elevated total bilirubin and liver enzymes, as well as a history of acholic stool and dark urine, the patient was admitted to general pediatrics for further work-up.

**Figure 5:**
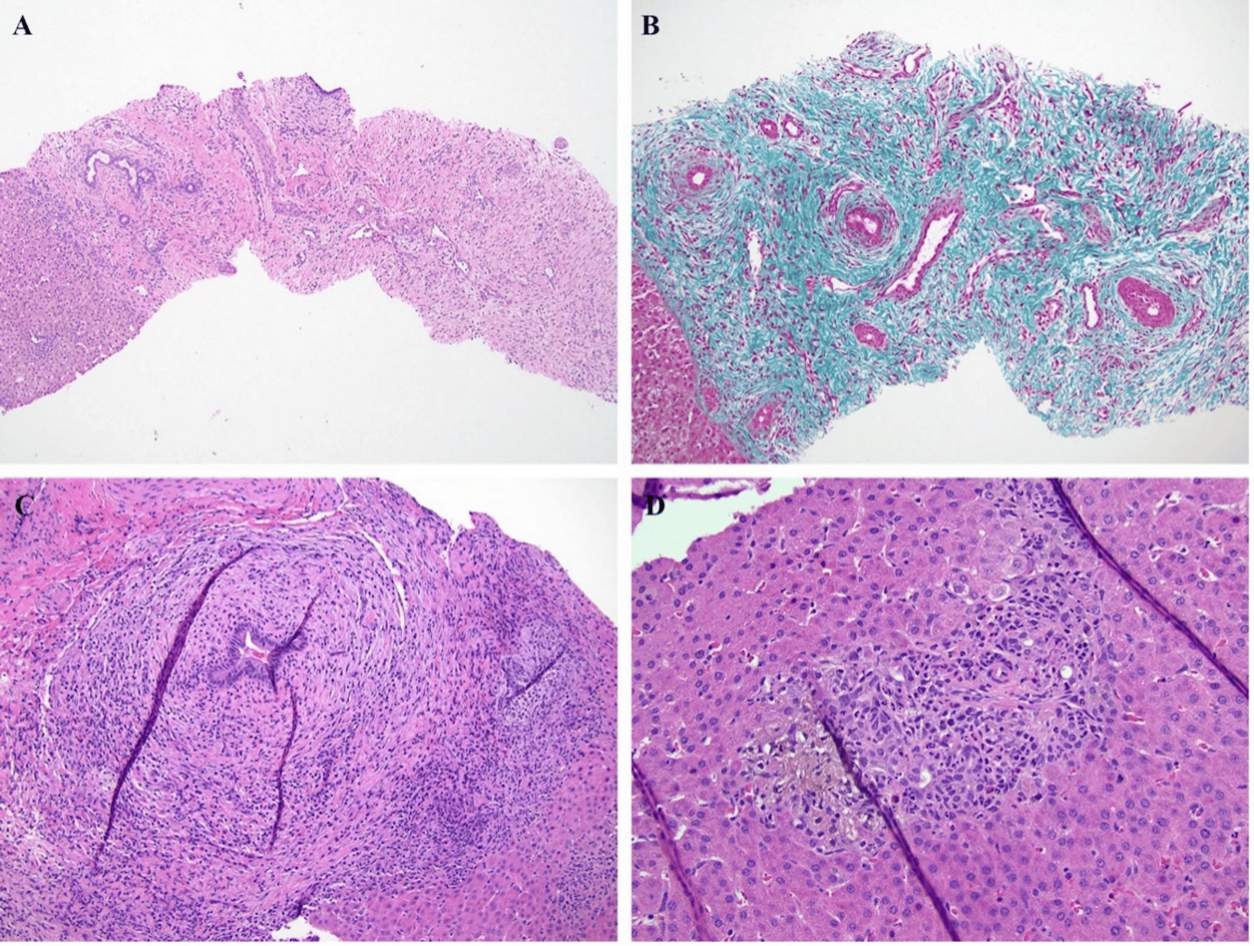
Histological findings for case presentation two are suggestive of acute and chronic large bile duct obstruction. (A, 4x) Liver biopsy shows marked periductal fibrous expansion surrounding a large bile duct, forming an "onion-skin" pattern. Mild periductal chronic inflammation and portal edema are also present. (B, 10x) Trichrome stain highlights the concentric periductal fibrosis. (C, 10x) A follow-up biopsy performed 12 days later shows persistent periductal edema, circumferential fibrosis of a large bile duct, and prominent bile ductular reactions. (D, 20x) A small bile lake is identified within the adjacent portal tract.

MRCP on hospital day two demonstrated abnormal filling with signal characteristics suggestive of a pigmented stone (Figure 4B, 4C). Liver biopsy on hospital day three demonstrated onion skin periductal fibrosis, which was morphologically consistent with chronic large duct obstruction. Ursodiol was prescribed to improve bile flow (Figure 5A, 5B). 

A second MRCP was performed on hospital day eight with continued biliary duct dilation, redemonstrating a filling defect in the distal CBD consistent with a pigmented gallstone. However, his AST, ALT, total bilirubin, and conjugated bilirubin were downtrending (Table 2). Additionally, his stools had returned to normal. He continued to show clinical improvement and he was discharged with close follow-up, as it was thought that he had passed the stone. At scheduled follow-up visit six days after discharge, his AST, ALT, total bilirubin, and conjugated bilirubin had all increased. 

A multidisciplinary team meeting was held with gastroenterology, radiology, and pediatric surgery, and the decision was made to pursue operative management because the patient was not a candidate for ERCP due to his size. In the operating room, a cholangiogram was performed, confirming the presence of a stone in the distal CBD. The gallbladder and cystic duct were opened to the level of the confluence with the CBD, and attempts were made to remove the stone with forceps, which were unsuccessful. An endoscope was passed into the CBD, and there was debris and webs within the distal CBD. Due to the presence of webs, the decision was made to perform a Roux-en-Y hepaticojejunostomy. Pathology of the intraoperative liver biopsy showed periductal edema, fibrosis, mild and chronic inflammation, and a periportal bile lake as well as acute and chronic large duct obstruction (Figure 5C, 5D). The gallbladder pathology demonstrated congestion with acute and chronic cholecystitis. The patient’s postoperative course was uncomplicated. He was discharged on post-operative day four with a 10-day course of trimethoprim-sulfamethoxazole [11]. In clinic two weeks after discharge, he had increased appetite, regular bowel movements and appropriate weight gain. His AST, ALT, total bilirubin, and conjugated bilirubin had all downtrended (Table 2). 

Genetic sequencing revealed a variant of unknown significance in Notch 2 and ABCB4 genes. The ABCB4 gene is related to low phospholipid-associated cholelithiasis syndrome, which is known to cause recurrence of biliary symptoms after cholecystectomy and hyperechoic intrahepatic foci or comet tail images within intrahepatic ducts [14].

## Discussion

Neonatal hyperbilirubinemia is most commonly physiologic and self-limited but may also represent underlying pathology [4]. The differential diagnosis for neonatal jaundice is first categorized based on unconjugated or conjugated hyperbilirubinemia. Unconjugated hyperbilirubinemia can be physiologic due to the increased production and decreased excretion of bilirubin in the newborn period and is more commonly seen in breastfed and premature infants. Less common causes include hemolysis, metabolic disorders, or diseases such as Gilbert’s and Crigler-Najjar. Physiologic jaundice is distinguished from pathologic causes by the timeline. Physiologic jaundice typically reaches a peak of total serum bilirubin levels between three to five days of life. Pathologic jaundice has an elevated peak value and a slower decline [4]. If there is suspicion for pathologic unconjugated hyperbilirubinemia, history, physical exam and laboratory tests are useful for eliciting the underlying cause such as ABO incompatibility, birth trauma, red blood cell structural defects, and others [4].

In contrast, elevations of conjugated bilirubin are always pathologic. In cases where bilirubin elevations persist beyond the expected immediate post-natal period, clinicians should have a low threshold for obtaining a fractionated bilirubin which reports both a conjugated and unconjugated bilirubin level. Further workup for neonatal conjugated hyperbilirubinemia should include testing for liver enzymes, infectious etiologies, such as CMV, hepatitis A, B, C, and E, as well as stool assessment and an alpha-1-antitrypsin assay. Newborn screenings evaluate for congenital hypothyroidism or galactosemia [4]. The first imaging test should be an ultrasound of the liver, gallbladder, biliary tree, and spleen. This is especially important for assessing for an abnormal gallbladder, absent common bile duct, or the triangular cord sign that can be seen in biliary atresia. Choledochal cysts can be identified on ultrasound as well. If suspicion for biliary atresia remains high, further work-up involving hepatobiliary scintigraphy, MRCP, and ERCP biopsy can be completed. Early diagnosis of biliary atresia is essential because Kasai procedures are more successful if performed earlier [4].

As seen in our cases, choledocholithiasis can be recognized from abdominal ultrasound as well. The neonates in this case study had no known contributory past medical history at the time that they were diagnosed, so a broad differential and the use of fractionated bilirubin, ultrasound and MRCP as diagnostic adjuncts was necessary. In both cases, the abdominal ultrasound was essential for narrowing the differential and MRCP was required to confirm the diagnoses and rule out other abnormalities of the biliary tree such as a choledochal cyst. Although choledocholithiasis is rare in neonates, it is important to identify and manage early. If left untreated, patients are at risk for serious complications, such as cholangitis, sepsis, gallstone pancreatitis, and cirrhosis [6].

Neonatal choledocholithiasis is a challenging diagnosis to manage, as patients are often unable to receive ERCP. Our cases describe the successful treatment of two infants with open cholecystectomy and Roux-en-Y hepaticojejunostomy. Further studies are needed to evaluate best practices for managing choledocholithiasis in this age group. Unlike adult patients, there are no standard guidelines for the management of choledocholithiasis in neonates. The three primary treatment options for adults described in the literature are ERCP with duct clearance, open surgical common bile duct exploration with cholecystectomy, and laparoscopic bile duct exploration with cholecystectomy [2]. ERCP provides a valuable, minimally invasive option for treating choledocholithiasis [15]. It has been utilized in the pediatric population, with increasing reports describing its success in children and babies under six months of age [15]. However, large retrospective studies of ERCP in the pediatric population show that while there are larger numbers of pediatric patients undergoing successful treatment with ERCP, there is significantly less data about the success of ERCP in infants [15]. The use of ERCP is limited in infants because the equipment is often not the appropriate size, the procedure is technically challenging to perform, and there are ongoing issues with sterilization processes for small endoscopes in North America [15-17]. Furthermore, while pediatric duodenoscopes are available, there are limitations to the size of biliary dilation balloons and stents that can be utilized through the 2-mm working channel [6]. Keil et al. describe limitations of ERCP for babies under 12 months of age because the duodenoscope cannot be used for papilla sphincterotomy. In their study, they placed a stent and treated non-operatively with ursodeoxycholic acid, then waited until the child was 12 months of age to remove the stent and perform the papilla sphincterotomy [16]. Additionally, there are complications associated with ERCP in pediatric patients, similar to the complications seen in adults, including pancreatitis, gastrointestinal bleeding, perforation, and cholangitis [15].

In settings where ERCP is not an option, surgery should be considered. This includes laparoscopic or open common bile duct exploration with cholecystectomy [3]. Reid et al. compared initial management with laparoscopic surgery versus ERCP in pediatric patients. They found that initial laparoscopic CBD exploration and cholecystectomy resulted in a decreased length of stay, decreased time to definitive intervention, and fewer procedures [18]. In our cases, ERCP was considered for both patients, but deemed not possible, and ultimately neither stone could be removed during open exploration due to the degree of stone impaction within the CBD. Open CBD exploration is technically challenging, and there are many reports of failure to retrieve a stone at the time of exploration [19]. In both of our cases, the patients underwent a Roux-en-Y hepaticojejunostomy.

The limited availability of ERCP equipment poses a significant challenge for treating choledocholithiasis in neonates. In North America, it is difficult to adequately sterilize duodenoscopes because the current reprocessing methods are not adequate for preventing reinfection, and solutions such as single scope duodenoscopes have associated issues, such as increased waste and expense [17]. Furthermore, in low- and middle-income countries, ERCP is often not available due to limitations with cost, maintenance, sterilization, and lack of qualified personnel to perform ERCP [20]. In infants with choledocholithiasis receiving treatment in these countries, surgical treatment may be the only option for management to prevent damage to the liver parenchyma.

## Conclusions

We present two cases of neonatal choledocholithiasis, managed with CBD exploration, cholecystectomy and Roux-en-Y hepaticojejunostomy. Choledocholithiasis remains a rare diagnosis in neonates, and both of our patients had genetic changes predisposing them to the condition. However, it is important for choledocholithiasis to be on the list of differential diagnoses for neonates with elevated conjugated bilirubin levels and/or elevated liver enzymes. If not identified, there are significant sequelae, such as cholangitis, that can occur. Ultrasound of the liver, gallbladder, biliary tree, and spleen can be utilized to identify choledocholithiasis and other anatomic causes of hyperbilirubinemia. Although ERCP has become the standard for management of choledocholithiasis in most adult and pediatric patients, ERCP is often not an option in infants due to the small size of the CBD and non-availability of small endoscopes. This is especially true in low- and middle-income countries. Therefore, it is important to describe other management strategies for neonatal choledocholithiasis that can be safely employed. We demonstrate the management of two patients who were not candidates for ERCP and who had unsuccessful CBD explorations. Both patients were successfully treated with a Roux-en-Y hepaticojejunostomy. While many strategies for neonatal choledocholithiasis have been described in the literature, there are not currently published guidelines. There is a need for established guidelines and improved equipment availability for managing choledocholithiasis in neonates.
